# The Journal of Cachexia, Sarcopenia and Muscle in 2019

**DOI:** 10.1002/jcsm.12482

**Published:** 2019-08-27

**Authors:** Stephan von Haehling, Nicole Ebner, Stefan D. Anker

**Affiliations:** ^1^ Department of Cardiology and Pneumology University of Göttingen Medical School, German Center for Cardiovascular Research (DZHK), partner site Göttingen Göttingen Germany; ^2^ Division of Cardiology and Metabolism—Heart Failure, Cachexia & Sarcopenia, Department of Cardiology (CVK), and Berlin‐Brandenburg Center for Regenerative Therapies (BCRT), Deutsches Zentrum für Herz‐Kreislauf‐Forschung (DZHK) Berlin Charité Universitätsmedizin Berlin Berlin Germany

The good news first: The *Journal of Cachexia, Sarcopenia and Muscle* (*JCSM*), now published in its 10th year, has nearly maintained last year's impact factor, even though it has decreased slightly, now reaching 10.754 as published by Thomson Scientific a few weeks ago. The impact factor is calculating from citations to the items in 2016 and 2017 divided by the number of items published in 2016 and 2017. It is important to understand that only the last full years count towards the impact factors, and this is why a journal that has been launched only 12 or 18 months ago can never have an impact factor even though some do calculate their ‘unofficial impact factor’, which should not be taken too seriously. In addition, it is important to note that the impact factor published in 2019 is the official 2018 impact factor, looked at and calculated using the citations and items published in the previous 2 years. So, at the time of this writing in July 2019, there is no 2019 impact factor, because this will only be published in 2020, usually in June. With regard to our own journal, we had total of 603 citations in 2016 and 838 in 2017. So that in total, 1441 citations were divided by 134 items published in these 2 years resulting in the calculated impact factor of 10.754.^1^ Fortunately, this places *JCSM* as the number 9 ranked journal among all journals in ‘Medicine: General and Internal’ (*Table* 1) and as the number 2 ranking journal among all nutrition journals, among which, however, *JCSM* is still not officially listed by Thomson Scientific. However, *JCSM* is listed in the category ‘Gerontology’, and it still reaches the top position there (*Table* 2).

**Table 1:** Top 10 journals in the field ‘Medicine: General and Internal’

	Journal name	Impact factor 2018	Items published 2016 and 2017	Issues per year
1	New England Journal of Medicine	70.670	655	52
2	Lancet	59.102	639	52
3	*Journal of the American Medical Association* (*JAMA*)	51.273	421	48
4	*Nature Reviews Disease Primers*	32.274	95	0
5	*British Medical Journal*	27.604	361	52
6	*JAMA Internal Medicine*	20.768	267	12
7	*Annals of Internal Medicine*	19.315	279	24
8	*PLOS Medicine*	11.048	393	12
9	*Journal of Cachexia, Sarcopenia and Muscle*	10.754	134	4
10	*BMC Medicine*	8.285	355	0

**Table 2:** Top Ten Journals in the field “Geriatrics” where the Journal of Cachexia, Sarcopenia and Muscle is officially listed.

	Journal name	Impact factor 2018	Items published 2016 and 2017	Issues per year
1	*Journal of Cachexia Sarcopenia and Muscle*	10.754	134	4
2	*Ageing Research Reviews*	10.390	172	4
3	*Aging Cell*	7.346	243	6
4	*GeroScience*	6.444	45	6
5	*Aging‐US*	5.515	363	12
6	*Journal of the American Medical Directors Association*	4.899	414	9
7	*Journals of Gerontology Series A‐Biological Sciences and Medical Sciences*	4.711	470	12
8	*Age*	4.648	88	6
9	*Age and Ageing*	4.511	280	6
10	*Neurobiology of Aging*	4.398	618	6

As before, we would like to sincerely thank all authors, reviewers, and editorial board members for their great efforts to make *JCSM* such a high‐quality and high‐ranked journal, and we greatly appreciate and value the interest and support of all those who enjoy reading *JCSM* and citing the papers published there.

This year is unique for *JCSM* in several ways. The most important is the transfer of the two daughter journals of *JCSM* to Wiley as a publishing house. Indeed, *JCSM* appears to have sparked more scientific interest in the field of body wasting, cachexia, and sarcopenia, and thus, the number of submissions to the main journal remains on the increase. With a 73% rejection rate—knowing that we have to decline publication of many good papers simply for lack of space—we hope to be able to give some of these a home in our two daughter journals—*JCSM Rapid Communications* and *JCSM Clinical Reports*. *JCSM Clinical Reports* is online since December 2016 and *JCSM Rapid Communications* since January 2018. The first is dedicated to publishing clinical studies from the area of cachexia, wasting, and muscle disorders, and the second aims to publish both clinical and basic research papers with high relevance to the audience in this still niche area. We hope that the three journals will help in clinical decision making and in serving as a source of clinical information and case reports as well.

The main journal, *JCSM*, has, at the time of this writing, received 294 submissions in 2019 alone. Last year at this time, we had received 209. We are working hard to provide a timely peer review, which is not always easy, as it is difficult at times to find appropriate reviewers. Articles that are available for the longest time are—not surprisingly—those that have been cited the most (*Table* 3). Our editorials remain popular (*Table* 4), and we invite our readers to submit their work or to suggest topics for ‘facts and numbers’ editorials that are relevant to our readers.

**Table 3:** Top 30 of best cited articles since first publication of the Journal of Cachexia, Sarcopenia and Muscle

Rank	First author	Title	Type	Year	Times cited	Reference
1	von Haehling	Cachexia as a major underestimated and unmet medical need: facts and numbers	Editorial	2010	385	2
2	Dalton	The selective androgen receptor modulator GTx‐024 (enobosarm) improves lean body mass and physical function in healthy elderly men and postmenopausal women: results of a double‐blind, placebo‐controlled phase II trial	Original article	2011	154	3
3	Morley	Prevalence, incidence, and clinical impact of sarcopenia: facts, numbers, and epidemiology—update 2014	Editorial	2014	144	4
4	Fanzani	Molecular and cellular mechanisms of skeletal muscle atrophy: an update	Review	2012	121	5
5	Lenk	Skeletal muscle wasting in cachexia and sarcopenia: molecular pathophysiology and impact of exercise training	Review	2010	117	6
6	Wakabayashi	Rehabilitation nutrition for sarcopenia with disability: a combination of both rehabilitation and nutrition care management	Review	2014	115	7
7	Cesari	Biomarkers of sarcopenia in clinical trials—recommendations from the International Working Group on Sarcopenia	Original article	2012	115	8
8	Morley	From sarcopenia to frailty: a road less traveled	Editorial	2014	114	9
9	Elkina	The role of myostatin in muscle wasting: an overview	Review	2011	111	10
10	von Haehling	An overview of sarcopenia: facts and numbers on prevalence and clinical impact	Editorial	2010	111	11
11	Mak	Wasting in chronic kidney disease	Review	2011	105	12
12	von Haehling	Prevalence, incidence and clinical impact of cachexia: facts and numbers—update 2014	Editorial	2014	104	13
13	Patel	Serum creatinine as a marker of muscle mass in chronic kidney disease: results of a cross‐sectional study and review of literature	Review	2013	103	14
14	Malstrom	SARC‐F: a symptom score to predict persons with sarcopenia at risk for poor functional outcomes	Original article	2016	101	15
15	Dasarathy	Consilience in sarcopenia of cirrhosis	Review	2012	93	16
16	Bowen	Skeletal muscle wasting in cachexia and sarcopenia: molecular pathophysiology and impact of exercise training	Review	2015	92	17
17	Vaughan	Cancer cachexia: impact, mechanisms and emerging treatments	Review	2013	82	18
18	von Haehling	From muscle wasting to sarcopenia and myopenia: update 2012	Editorial	2012	80	19
19	Lainscak	The obesity paradox in chronic disease: facts and numbers	Editorial	2012	80	20
20	Lainscak	Body mass index and prognosis in patients hospitalized with acute exacerbation of chronic obstructive pulmonary disease	Original article	2011	79	21
21	Fearon	Myopenia—a new universal term for muscle wasting	Editorial	2011	73	22
22	Montano‐Loza	Sarcopenic obesity and myosteatosis are associated with higher mortality in patients with cirrhosis	Original article	2016	72	23
23	Calvani	Biomarkers for physical frailty and sarcopenia: state of the science and future developments	Review	2015	72	24
24	Rozentryt	The effects of a high‐caloric protein‐rich oral nutritional supplement in patients with chronic heart failure and cachexia on quality of life, body composition, and inflammation markers: a randomized, double‐blind pilot study	Original article	2010	70	25
25	Schefold	Intensive care unit‐acquired weakness (ICUAW) and muscle wasting in critically ill patients with severe sepsis and septic shock	Review	2010	69	26
26	Chen	Ghrelin prevents tumour‐ and cisplatin‐induced muscle wasting: characterization of multiple mechanisms involved	Original article	2015	68	27
27	Anker	Welcome to the ICD‐10 code for sarcopenia	Editorial	2016	65	28
28	Hexmsfield	Assessing skeletal muscle mass: historical overview and state of the art	Review	2014	65	29
29	Farkas	Cachexia as a major public health problem: frequent, costly, and deadly	Review	2013	64	30
30	Santarpia	Butyrylcholinesterase as a prognostic marker: a review of the literature	Review	2013	64	31

**Table 4:** Top 30 articles published in 2016/17 sorted by citations in 2018. Of note, these 30 papers contributed 36% of citations for our impact factor 2018

Rank	First author	Title	Type	Year	Times cited	Reference
1	Malmstrom, Theodore K.	SARC‐F: a symptom score to predict persons with sarcopenia at risk for poor functional outcomes	Original article	2016	36	15
2	Montano‐Loza, Aldo J.	Sarcopenic obesity and myosteatosis are associated with higher mortality in patients with cirrhosis	Original article	2016	29	23
3	Rutten, Iris J. G.	Loss of skeletal muscle during neoadjuvant chemotherapy is related to decreased survival in ovarian cancer patients	Original article	2016	27	32
4	van Dijk, David P. J.	Low skeletal muscle radiation attenuation and visceral adiposity are associated with overall survival and surgical site infections in patients with pancreatic cancer	Original article	2017	24	33
5	Tyrovolas, Stefanos	Factors associated with skeletal muscle mass, sarcopenia, and sarcopenic obesity in older adults: a multi‐continent study	Original article	2016	22	34
6	Kalafateli, Maria	Malnutrition and sarcopenia predict post‐liver transplantation outcomes independently of the Model for End‐stage Liver Disease score	Original article	2017	21	35
7	Boengler, Kerstin	Mitochondria and ageing: role in heart, skeletal muscle and adipose tissue	Review	2017	21	36
8	Brown, Justin C.	Sarcopenia and mortality among a population‐based sample of community‐dwelling older adults	Original article	2016	20	37
9	Solheim, Tora S.	A randomized phase II feasibility trial of a multimodal intervention for the management of cachexia in lung and pancreatic cancer	Original article	2017	19	38
10	Loncar, Goran	Cardiac cachexia: hic et nunc	Review	2016	18	39
11	Nijholt, Willemke	The reliability and validity of ultrasound to quantify muscles in older adults: a systematic review	Review	2017	17	40
12	Mochamat, Henning C.	A systematic review on the role of vitamins, minerals, proteins, and other supplements for the treatment of cachexia in cancer: a European Palliative Care Research Centre cachexia project	Review	2017	17	41
13	Rutten, Iris J. G.	Psoas muscle area is not representative of total skeletal muscle area in the assessment of sarcopenia in ovarian cancer	Original article	2017	17	42
14	Leong, Darryl P.	Reference ranges of handgrip strength from 125,462 healthy adults in 21 countries: a prospective urban rural epidemiologic (PURE) study	Original article	2016	16	43
15	Holecek, Milan	Beta‐hydroxy‐beta‐methylbutyrate supplementation and skeletal muscle in healthy and muscle‐wasting conditions	Review	2017	16	44
16	Sanders, Karin J. C.	Cachexia in chronic obstructive pulmonary disease: new insights and therapeutic perspective	Review	2016	15	45
17	Reijnierse, Esmee M.	Assessment of maximal handgrip strength: how many attempts are needed?	Original article	2017	15	46
18	Stewart Coats, Andrew J.	Espindolol for the treatment and prevention of cachexia in patients with stage III/IV non‐small cell lung cancer or colorectal cancer: a randomized, double‐blind, placebo‐controlled, international multicentre phase II study (the ACT‐ONE trial)	Original article	2016	14	47
19	Kittiskulnam, Piyawan	Sarcopenia among patients receiving hemodialysis: weighing the evidence	Original article	2017	14	48
20	Snijders, Tim	Muscle fibre capillarization is a critical factor in muscle fibre hypertrophy during resistance exercise training in older men	Original article	2017	14	49
21	Foong, Yi Chao	Accelerometer‐determined physical activity, muscle mass, and leg strength in community‐dwelling older adults	Original article	2016	13	50
22	Nishikawa, Hiroki	Elevated serum myostatin level is associated with worse survival in patients with liver cirrhosis	Original article	2017	13	51
23	Martone, Anna Maria	The incidence of sarcopenia among hospitalized older patients: results from the Glisten study	Original article	2017	13	52
24	van de Bool, Coby	A randomized clinical trial investigating the efficacy of targeted nutrition as adjunct to exercise training in COPD	Original article	2017	13	53
25	Clark, Andrew L.	Effect of beta‐adrenergic blockade with carvedilol on cachexia in severe chronic heart failure: results from the COPERNICUS trial	Original article	2017	13	54
26	van Vugt, Jeroen L. A.	A comparative study of software programmes for cross‐sectional skeletal muscle and adipose tissue measurements on abdominal computed tomography scans of rectal cancer patients	Original article	2017	13	55
27	Beaudart, Charlotte	Validation of the SarQoL^®^, a specific health‐related quality of life questionnaire for sarcopenia	Original article	2017	13	56
28	Barbosa‐Silva, Thiago G.	Prevalence of sarcopenia among community‐dwelling elderly of a medium‐sized South American city: results of the *COMO VAI?* study	Original article	2016	13	57
29	Batista, Miguel L., Jr.	Cachexia‐associated adipose tissue morphological rearrangement in gastrointestinal cancer patients	Original article	2016	13	58
30	Nederveen, Joshua P.	Skeletal muscle satellite cells are located at a closer proximity to capillaries in healthy young compared with older men	Original article	2016	13	59

Finally, we would like to draw your attention to the upcoming Cachexia Conference, to be held between December 6 and 8, 2019, in Berlin, Germany. The conference became an annual meeting, and it is a source of stimulating ideas and exchange between clinicians and researchers in the field of cachexia and wasting. More information can be found at the following link: http://society‐scwd.org.

## Conflict of interests

The authors declare that no conflict of interest relevant to this article exists.
